# Irritable bowel syndrome and Parkinson’s disease risk: register-based studies

**DOI:** 10.1038/s41531-020-00145-8

**Published:** 2021-01-05

**Authors:** Bojing Liu, Arvid Sjölander, Nancy L. Pedersen, Jonas F. Ludvigsson, Honglei Chen, Fang Fang, Karin Wirdefeldt

**Affiliations:** 1 Department of Medical Epidemiology and Biostatistics, Karolinska Institutet, Stockholm, Sweden; 2 Department of Psychology, University of Southern California, Los Angeles, CA USA; 3 Department of Paediatrics, Örebro University Hospital, Örebro, Sweden; 4 Division of Epidemiology and Public Health, School of Medicine, University of Nottingham, Nottingham, UK; 5 Celiac Disease Center, Department of Medicine, Columbia University College of Physicians and Surgeons, New York, NY USA; 6 Department of Epidemiology and Biostatistics, College of Human Medicine, Michigan State University, East Lansing, MI USA; 7 Institute of Environmental Medicine, Karolinska Institutet, Stockholm, Sweden; 8 Department of Clinical Neuroscience, Karolinska Institutet, Stockholm, Sweden

**Keywords:** Parkinson's disease, Epidemiology

## Abstract

To examine whether irritable bowel syndrome (IBS) was related to the future risk of Parkinson’s disease (PD), we conducted a nested case-control study in the Swedish total population including 56,564 PD cases identified from the Swedish Patient Register and 30 controls per case individually matched by sex and year of birth. Odds ratios (ORs) with 95% confidence intervals (CIs) for having a prior diagnosis of IBS were estimated using conditional logistic regression. We furthermore conducted a cohort study using the Swedish Twin Registry following 3046 IBS patients identified by self-reported abdominal symptoms and 41,179 non-IBS individuals. Through Cox proportional hazard models, we estimated hazard ratios (HRs) and 95% CIs for PD risk. In the nested case-control study, 253 (0.4%) PD cases and 5204 (0.3%) controls had a previous IBS diagnosis. IBS diagnosis was associated with a 44% higher risk of PD (OR = 1.44, 95% CI 1.27–1.63). Temporal relationship analyses showed 53% and 38% increased risk of PD more than 5 and 10 years after IBS diagnosis, respectively. In the cohort analysis based on the Swedish Twin Registry, there was no statistically significantly increased risk of PD related to IBS (HR = 1.25, 95% CI = 0.87–1.81). Our results suggest a higher risk of PD diagnosis after IBS. These results provide additional evidence supporting the importance of the gut–brain axis in PD.

## Introduction

Parkinson’s disease (PD) is a neurodegenerative disease defined by the cardinal motor symptoms tremor, bradykinesia, rigidity, and postural imbalance^1^. PD is characterized by selective loss of dopaminergic neurons in the *substantia nigra pars compacta* and aggregations of α-synuclein in Lewy bodies and Lewy neurites^1^. The so-called gut–brain axis and associated inflammatory conditions have gained much attention in the etiology and pathogenesis of PD^2^. Although still controversial, Braak’s hypothesis suggested that, in some PD patients, the pathology may first start in the enteric nervous system (ENS) and later spread to the central nervous system (CNS) via the vagus nerve^3–5^. In line with this, constipation can occur prior to PD motor symptoms by years^6,7^. Aggregated α-synuclein has also been detected in the ENS of prodromal PD cases^8^. Interestingly, epidemiological studies have found that truncal vagotomy, which interrupts the gut–brain axis via the vagus nerve, was associated with lower PD risk^9,10^.

Although potential mechanisms for the gut–brain-interaction in PD etiology remain unclear, it may involve intestinal inflammation, intestinal permeability, and gut microbiota^2,11^. One hypothesis is that gastrointestinal (GI) inflammatory triggers disturb the composition of gut microbiota, increase intestinal permeability, and induce α-synuclein aggregation in the ENS, which is later retrogradely transported to the CNS^2^. Alternatively, inflammation and altered permeability in the gut promote chronic systemic inflammation and increase the blood–brain barrier permeability, which in turn may contribute to neuroinflammation and neurodegeneration in PD^2^.

This evidence prompts the investigations on GI diseases and PD risk. Irritable bowel syndrome (IBS) is a functional bowel disorder characterized by recurrent abdominal pain or discomfort and altered bowel habits^12,13^. IBS is categorized into three subtypes according to the predominant stool patterns, IBS-diarrhea, IBS-constipation, and mixed IBS with both constipation and diarrhea^14^. The pathogenesis of IBS is complex and traditionally focused on motility, visceral sensation, dysfunction of the gut–brain axis, and psychological stress, while recent evidence has highlighted the involvement of altered gut microbiota, increased intestinal permeability, and immune activation, such as low-grade mucosal inflammation^12,13,15^.

Few studies have examined the relationship between IBS and PD risk. One cohort study using data from the Taiwan National Health Insurance program reported a 48% higher risk of PD for IBS patients^16^. However, the results are limited by short follow-up (<10 years), possible underdiagnosis of IBS, potential confounding by smoking and alcohol consumption, and surveillance bias. It is further unknown whether this potential association can be explained by constipation.

We aimed to examine the association between IBS and PD risk using two large complementary databases—the nationwide health and population registers and the Swedish Twin Registry. The first analysis capitalized the large sample size, while the second analysis had information on important potential confounders including constipation, smoking, and alcohol consumption. We hypothesized that IBS was associated with a higher risk of PD.

## Results

### Nationwide nested case-control study

Characteristics of PD cases and controls are displayed in Table 1. The distributions of sex and year of birth were the same in cases and controls by matching. Among the 56,564 PD cases, mean age (±SD) at index date was 75.1 ± 9.41 years (72.5 ± 9.97 for primary and 78.0 ± 7.79 for secondary PD cases). Being born outside of Sweden, having lower educational attainment, and having COPD were associated with lower PD risk, while having more comorbidities was associated with higher risk of PD.

**Table 1:** Characteristics of Parkinson’s disease (PD) cases and controls from Swedish total population 1987–2010, *N* = 1,753,484.

	PD cases, *N* (%)	Controls, *N* (%)	OR (95% CI)^a^
*Total*	56,564 (100)	1,696,920 (100)	–
*Sex*			
Male	31,807 (56.2)	954,210 (56.2)	–
Female	24,757 (43.8)	742,710 (43.8)	–
*Age at the index date, years*			
<60	3926 (6.9)	118,416 (7.0)	–
60–69	9863 (17.4)	295,717 (17.4)	–
70–79	24,164 (42.7)	724,883 (42.7)	–
≥80	18,611 (32.9)	557,904 (32.9)	–
*Born in Sweden*			
Unknown	1	103	–
No	3603 (6.4)	113,027 (6.7)	0.95 (0.92–0.99)
Yes	52,960 (93.6)	1,583,790 (93.3)	1
*Highest achieved education, years*			
Unknown	8170 (14.4)	245,218 (14.5)	0.88 (0.82–0.96)
≤9	26,537 (46.9)	821,795 (48.4)	0.87 (0.84–0.89)
10–12	14,782 (26.1)	437,942 (25.8)	0.91 (0.89–0.94)
>12	7075 (12.5)	191,965 (11.3)	1
*Chronic obstructive pulmonary disease (COPD)*			
No	55,244 (97.7)	1,648,443 (97.1)	1
Yes	1,320 (2.3)	48,477 (2.9)	0.81 (0.77–0.86)
*Comorbidity index*			
0	33,514 (59.2)	1,061,214 (62.5)	1
1–2	17,447 (30.8)	480,548 (28.3)	1.16 (1.14–1.19)
≥3	5,603 (9.9)	155,158 (9.1)	1.16 (1.13–1.20)

^a^Conditional on birth year and sex matching pairs.

A total of 5457 IBS patients were identified; 253 in the PD group and 5204 in the matched control group. Over the entire observational time, IBS was associated with 44% higher risk of PD (Table 2). In the temporal relationship analysis, we observed 53% elevated PD risk ≥5 years and 38% elevated PD risk ≥10 years after IBS diagnosis. The association appeared to be more prominent among IBS patients diagnosed at age 50 or older, although the test for interaction was not statistically significant (*p* for interaction = 0.11). Similar results were observed in men and women (*p* for interaction = 0.81) and different age groups at index date (*p* for interaction = 0.71). Additional adjustment for simulated smoking status did not change the results, while adjustment for simulated constipation status attenuated the results from HR = 1.46 to HR = 1.24 (95% CI = 1.10–1.40) (Supplementary Table 3). An *E*-value of 1.89 indicated that an unmeasured confounder, associated with both IBS and PD by relative risk of 1.89, would entirely explain the observed results between IBS and PD. Analyses restricting to primary register PD diagnosis and additionally adjusting for number of hospital visits generally yielded somewhat weaker associations (Supplementary Table 4). When restricting to primary PD diagnoses, we noted a 41% increased risk ≥5 years after IBS diagnosis. When adjusting for number of hospital visits, we found a 31% increased risk of PD ≥5 years after IBS.

**Table 2:** Irritable bowel syndrome (IBS) diagnosis and risk of Parkinson’s disease (PD), nationwide case-control analysis.

	PD cases	Controls	Model 1^a^	Model 2^b^
	*N*	*N*	OR (95% CI)	OR (95% CI)
*Non-IBS*	56,311 (99.6)	1,691,716 (99.7)	1	1
*IBS*	253 (0.4)	5204 (0.3)	1.46 (1.29–1.66)	1.44 (1.27–1.63)
Years before index date				
<5	93 (0.2)	2105 (0.1)	1.33 (1.08–1.64)	1.31 (1.06–1.61)
≥5	160 (0.3)	3099 (0.2)	1.55 (1.32–1.82)	1.53 (1.30–1.79)
<10	166 (0.3)	3331 (0.2)	1.50 (1.28–1.75)	1.47 (1.26–1.72)
≥10	87 (0.2)	1873 (0.1)	1.40 (1.13–1.73)	1.38 (1.11–1.71)
Age at IBS diagnosis, years				
<50	28 (0)	766 (0)	1.10 (0.75–1.60)	1.09 (0.74–1.58)
≥50	225 (0.4)	4438 (0.3)	1.52 (1.33–1.74)	1.50 (1.31–1.72)
*Stratified by sex*				
Male	89 (0.2)	1789 (0.1)	1.49 (1.21–1.85)	1.47 (1.19–1.82)
Female	164 (0.3)	3415 (0.2)	1.44 (1.23–1.69)	1.42 (1.22–1.67)
*Stratified by age on the index date, years*				
<70	80 (0.1)	1538 (0.1)	1.57 (1.25–1.96)	1.55 (1.24–1.94)
70–79	101 (0.2)	2184 (0.1)	1.39 (1.14–1.70)	1.36 (1.12–1.67)
≥80	72 (0.1)	1482 (0.1)	1.46 (1.15–1.85)	1.44 (1.13–1.82)

^a^Conditional on birth year and sex.

^b^Additionally adjusted for country of birth, highest achieved education, chronic obstructive pulmonary disease (COPD), comorbidity index.

### Cohort analysis in the Swedish Twin Registry

**Fig. 1:**
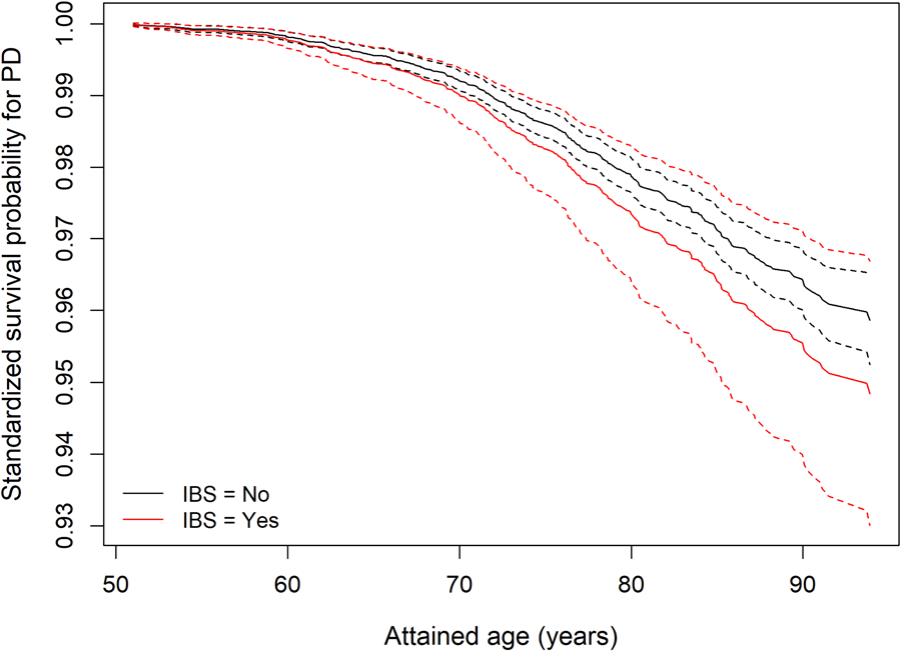
Standardized survival function for Parkinson’s disease. Standardized survival function and confidence interval for Parkinson’s disease (PD) comparing individuals with irritable bowel syndrome with those without in SALT Swedish twins. Standardized survival functions of PD were estimated and plotted using regression standardization after fitting the multivariable adjusted Cox model.

Characteristics of the twins in the *Screening Across the Lifespan of Twins Study* (SALT) are shown in Table 3. At baseline, we identified 3046 individuals with IBS and 41,179 persons without based on self-reported abdominal symptoms. Female sex, older age, higher education level, alcohol consumption, and ever smokers were more prevalent among people with IBS, while the comorbidity index was similar in IBS and non-IBS individuals. Mean age (±SD) at interview was 57.1 (±9.9) years for IBS and 59.5 (±11.1) years for non-IBS individuals. During an average of 15.1 (median 16.0) years of follow-up, 474 PD patients were identified. Mean age (±SD) at PD diagnosis was 73.6 ± 8.86 years. Crude incidence rate (per 10,000 person-years) for PD was 7.0 among IBS patients and 7.1 among non-IBS individuals. Although the HR estimate was above one, we found no statistically significant association between symptom-based IBS and PD risk (Table 4). Standardized survival curves indicated a higher PD risk among individuals with IBS compared to those without (Fig. 1). Parts of the results presented above were included in the first author’s doctoral thesis^17^.

**Table 3:** Characteristics of the cohort based on SALT Swedish twins, *N* = 44,225.

	IBS, *N* (%)	Non-IBS, *N* (%)	*p*-Value^a^
*Total*	3046 (100)	41,179 (100)	
*Sex*			<0.01
Male	1069 (35.1)	19,569 (47.5)	
Female	1977 (64.9)	21,610 (52.5)	
*Age at interview, years*			<0.01
<60	2047 (67.2)	24,041 (58.4)	
60–69	589 (19.3)	8785 (21.3)	
70–79	324 (10.6)	5950 (14.4)	
≥80	86 (2.8)	2403 (5.8)	
*Highest achieved education, years*			<0.01
Unknown	15 (0.5)	1493 (3.6)	
≤9	1352 (44.4)	19,203 (46.6)	
9–12	903 (29.6)	10,960 (26.6)	
≥12	776 (25.5)	9523 (23.1)	
*Smoking*			<0.01
Never	1111 (36.5)	16,640 (40.4)	
Ever	1919 (63.0)	23,010 (55.9)	
Unknown	16 (0.5)	1529 (3.7)	
*Alcohol consumption in the past month*			0.02
No	1057 (34.7)	13,135 (31.9)	
Yes	1703 (55.9)	23,355 (56.7)	
Missing	286 (9.4)	4689 (11.4)	
*Comorbidity index*			0.79
0	1413 (46.4)	18,983 (46.1)	
1–2	903 (29.6)	12,447 (30.2)	
≥3	730 (24.0)	9749 (23.7)	

^a^Chi-square test.

**Table 4:** Self-reported irritable bowel syndrome and Parkinson’s disease (PD) risk in SALT Swedish twins.

	PD case	Person-years	Model 1^a^ HR (95% CI)	Model 2^b^ HR (95% CI)
Non-IBS	441	618,513.0	1	1
IBS	33	47,195.2	1.17 (0.82–1.67)	1.25 (0.87–1.81)

^a^Adjusted for sex and attained age.

^b^Additionally adjusted for highest achieved education, smoking history, alcohol consumption in the last month, and comorbidity index.

## Discussion

We examined the association between IBS and subsequent PD risk in two complementary epidemiological databases, the nationwide health registers and the Swedish Twin Registry. In the nationwide nested case-control study, we observed higher PD risk both ≥5 and ≥10 years after specialist IBS diagnosis. Statistical significance of the associations remained in several sensitivity analyses. In the twin cohort, self-reported symptom-based IBS was not statistically significantly associated with PD risk.

Our results are consistent with a previous study that reported a 48% increased PD risk among IBS patients^16^. Similar to that study^16^, we did not notice any difference by sex, but the association appeared to be stronger for older (age at diagnosis ≥50 years) than younger (age at diagnosis <50 years) IBS patients. Cross-sectional studies also reported higher prevalence of IBS-like symptoms in PD patients compared to controls (i.e. 24.3% vs. 5.3%)^18^ or the general population (i.e. 17% vs. 13.1–14.0%)^19^. An important strength of our study is that we ascertained IBS in two complementary databases; we captured specialist diagnosed IBS patients by the Patient Register and IBS patients treated in primary care or who never sought care by the Twin Registry. IBS patients treated by gastroenterologists tend to have more severe symptoms than those who are only treated in primary care^20,21^. Presence of more severe symptoms has been linked to increased inflammatory response;^12^ with increased levels of several cytokines in serum or gut mucosa (e.g. interleukin −1β, −10, −6, and tumor necrosis factor α) and higher mast-cell counts^12^. Given the potential driving role of intestinal inflammation in PD, this may potentially explain that we observed a stronger association with PD for specialist diagnosed IBS than for self-reported symptom-based IBS.

Although mechanisms underlying the association between IBS and PD remain unknown, potential explanations for our results are shared etiology between the diseases or that IBS serves as a prodromal condition of PD. PD has a long prodromal phase, and non-motor symptoms can arise several decades prior to motor onset^22^. Altered intestinal permeability, intestinal inflammation, and disturbance of microbiome not only contribute to the pathogenesis of IBS^12^ but may also promote synucleinopathy in the enteric nerves, which may over time spread to the brain and eventually lead to PD pathology in the substantia nigra^2–5^. Increased intestinal permeability has been related to low-grade inflammation, pain, and diarrhea in IBS^23^. In newly diagnosed PD patients, intestinal permeability was significantly higher than in heathy controls and correlated with intestinal neuronal α-synuclein aggregates^24^. Intestinal inflammation has been suggested as a contributing factor in PD pathogenesis. For example, elevated inflammatory markers were found in stool samples of early PD patients^25^. Although inconsistent, evidence from epidemiological studies suggests that inflammatory bowel disease may be linked to PD risk^26,27^. Gut dysbiosis is involved in the pathophysiology of IBS^28^ and a study based on a mouse model of PD suggested that gut microbiota were required for promoting neuro-inflammation and α-synuclein mediated motor deficits^29^. Epidemiological studies reported dysbiosis in patients with PD^30–32^ as well as patients with rapid eye movement sleep behavior disorder—a strong predictor for PD^33^.

We performed specific analyses to control for different types of bias. As constipation is a major symptom in IBS, and there is converging evidence that constipation also is a prodromal symptom of PD^6,7^, we performed a sensitivity analysis adjusting for simulated constipation status in the nested-case control data. The association between IBS and PD remained, albeit attenuated, which indicates that constipation is unlikely to entirely explain our results. Further, the lag time between symptom onset and PD diagnosis in the Patient Register (on average 7.5 years for inpatient diagnoses based on a previous validation study^34^, likely shorter for outpatient diagnoses) raises the issue about potential reverse causation, such that PD results in IBS-like symptoms rather than vice versa. We addressed this by examining the long-term relationship between IBS and PD, showing a 38% increased PD risk more than 10 years after the IBS diagnosis^34^. Moreover, PD diagnosed shortly after IBS may be attributed to surveillance bias, whereby motor symptoms are detected as part of the clinical work-up for IBS. We controlled for surveillance bias by adjusting for number of hospital visits; the association was attenuated but did not disappear as a 31% higher PD risk remained ≥5 years after IBS diagnosis.

Our study has several strengths. With 1.7 million participants and long study period between 1964 and 2010, we had statistical power to examine the time-dependent relationship between IBS and future risk of PD. We captured IBS with various disease severity by identifying both gastroenterologist-diagnosed cases and symptom-based cases. We used a rigorous matching design and analyzed our data carefully with adjustment for several co-variates to reduce confounding and several sensitivity analyses to evaluate the robustness of our results. Finally, if the observed association is confirmed and proven to be causal, we expect that the results could be generalized to populations in other parts of the world.

The study also has some limitations. First, underdiagnosis and misclassification of IBS are inevitable in both the Patient Register and the Swedish Twin Registry and may potentially bias the results toward unexpected directions. Second, measurement error in date of IBS and PD diagnosis may be substantial, which limits the importance of the temporal relationship analysis. Third, due to lack of information in the Patient Register, we were not able to control for potential confounding by lifestyle factors and medication use and therefore we cannot rule out residual confounding as an alternative explanation to the results. Finally, results based on Swedish twins had large statistical uncertainty and we were not able to perform analyses within twin pairs due to small numbers.

In conclusion, our data suggest that IBS may be associated with an increased risk of PD and provide additional evidence supporting the importance of the gut–brain axis in PD etiology. Further investigations are needed to confirm the results in other populations and to address the potential mechanisms underlying the relationship.

## Methods

### Swedish health and population registers

The Swedish *Patient Register* was established in 1964–1965 and became nationwide in 1987. It collects information on dates of admission and discharge of hospitalizations, surgical procedures, and medical diagnoses^35,36^. The Patient Register was expanded to cover surgical day-care procedures in 1997 and outpatient visits in 2001^37^. For each hospital discharge or outpatient visit, a primary diagnosis and any secondary diagnoses are recorded. The *Causes of Death Register* contains nationwide information on deaths since 1952, and the *Total Population Register* holds information on dates of immigration and emigration^38^. The Swedish *Population and Housing Censuses* were conducted every 5 years from 1960 to 1990 to collect detailed information on housing, civil status, educational attainment, income, occupation, and social class^39^.

The Swedish *Twin Registry* is a nationwide population-based twin register established in the late 1950s. In SALT, telephone interviews were conducted during 1998–2002 with twins born before 1958^40,41^. During these interviews, information was collected regarding a broad spectrum of diseases and conditions, medication use, life style factors (e.g. smoking, alcohol consumption, and diet), occupation, and education^40,41^.

### Ascertainment of PD

We identified PD cases from the Patient Register using Swedish revisions of International Classification of Diseases (ICD) codes (i.e. ICD-7: 350 in 1964–1968; ICD-8: 342.00 in 1969–1986; ICD-9: 332.0 in 1987–1996; and ICD-10: G20 from 1997 onward). Both primary and secondary PD diagnoses were considered. We previously validated the inpatient PD diagnoses against clinical work-up as gold standard; the positive predictive value (PPV) was 70.8% for any primary or secondary PD diagnosis and the PPV increased to 80.3% when restricted to primary PD diagnosis^34^.

### Study design

In the present study, we applied two different study designs: (1) a nested case-control study based on the linkage of several Swedish nationwide registers via the unique Swedish personal identity number, and (2) a cohort study based on the Swedish Twin Registry.

Nationwide nested case-control study: Based on the Population and Housing Census in 1980, we conducted a nested case-control study in the total population of Sweden, who were born between 1889 and 1980 without previous PD diagnosis, and lived in Sweden on January 1, 1987 (*N* = 7,557,897). PD patients were identified between January 1, 1987, and December 31, 2010, by linkage to the Patient Register. For each PD patient, we randomly selected 30 controls, who were alive and living in Sweden, currently without PD diagnosis, individually matched to the case by sex and year of birth, on the date of PD diagnosis. The date of PD diagnosis was hereafter referred to as the index date for both PD cases and controls. In total, 56,564 PD cases and 1,696,920 controls were included in the nested case-control study.

Cohort study in the Swedish Twin Registry: The cohort study was based on all twins who responded to the SALT interview carried out in 1998–2002 (*N* = 44,919). Twins who discontinued participation with missing information on linkage or the date of emigration, or errors on date of death (*N* = 69), or had a PD diagnosis identified from the Patient Register or by self-report before or at the interview (*N* = 96), or had missing information on IBS at the interview (*N* = 529) were excluded. In total, the study population included 44,225 twins, who were followed from the date of interview until date of PD diagnosis, death, emigration out of Sweden, or December 31, 2016, whichever occurred first.

### Ascertainment of IBS

Nationwide nested case-control study: IBS cases were identified as individuals with a primary or secondary IBS diagnosis in the Patient Register according to the Swedish revisions of ICD codes (ICD-7: 573.10, 573.21, 573.22 in 1964–1968; ICD-8: 564.10, 564.11, 564.19 in 1969–1986; ICD-9: 564B in 1987–1996; and ICD-10: K58 from 1997 onward). We excluded IBS with previous diagnoses of inflammatory bowel disease, celiac disease, or colorectal cancer in the Patient Register (see ICD codes in Supplementary Table 1).

**Fig. 2:**
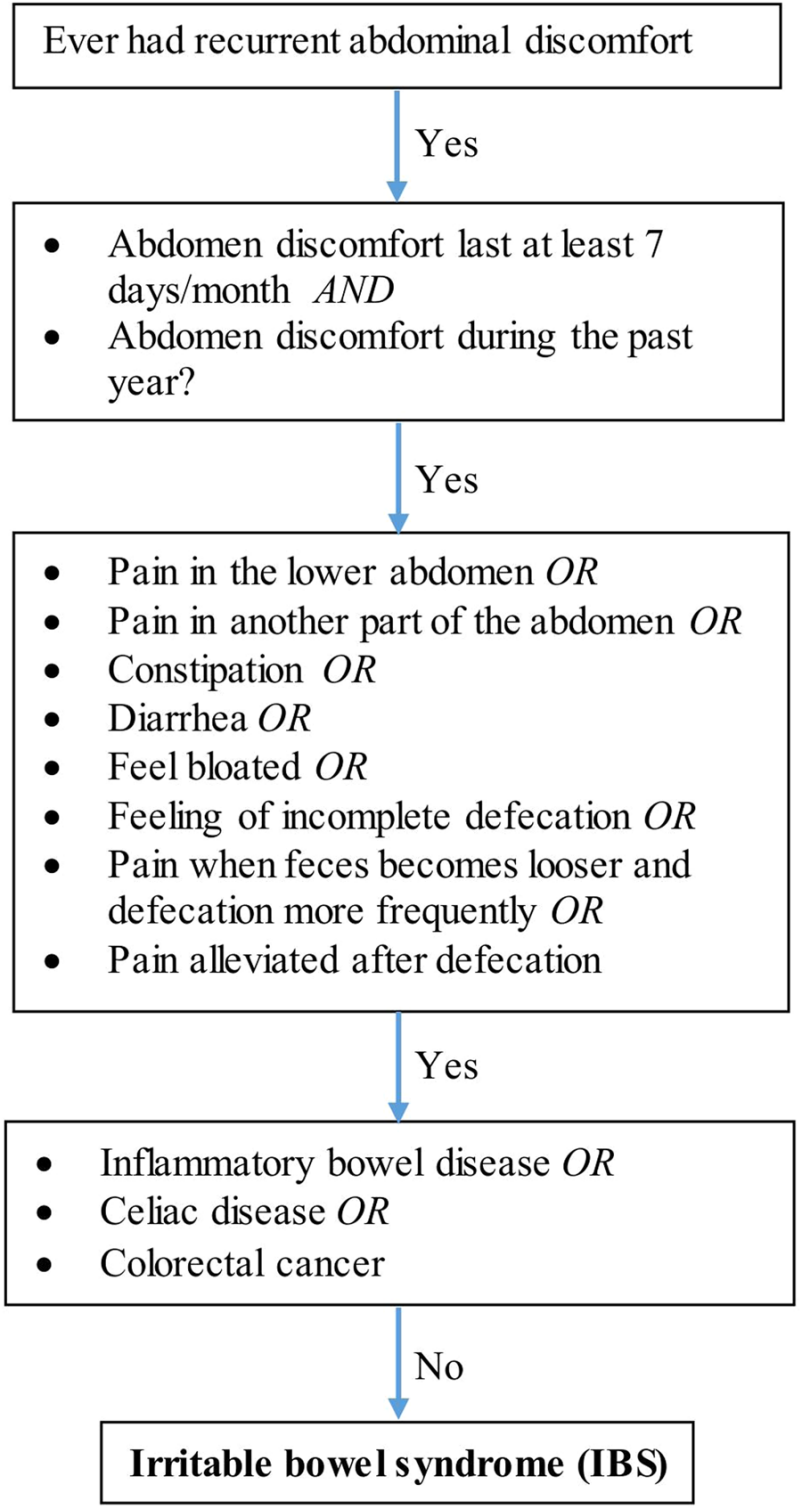
Definition of irritable bowel syndrome in SALT Swedish twins. Self-reported symptoms used to define the presence of irritable bowel syndrome (IBS) in SALT Swedish twins.

Cohort study in the Swedish Twin Registry: In SALT, we defined IBS patients using an algorithm involving self-reported abdominal symptoms based on the ROME II criteria^42^, which were the most recent criteria when the interviews were performed (Fig. 2). The algorithm has been validated^42^ against clinical IBS diagnosis based on the Rome II criteria, reliability of the algorithm to identify IBS was high, with a kappa coefficient of 0.92 and concordance of 99%^42^. We excluded patients who had previous diagnoses of inflammatory bowel disease, celiac disease, or colorectal cancer identified from the Patient Register.

### Covariates

Nationwide nested case-control study: Age at index date was categorized as ≤59, 60–69, 70–79, or ≥80 years. Information on country of birth was retrieved from the Total Population Register and participants were defined as either Swedish or non-Swedish born. Educational attainment was retrieved from the Swedish Register of Education and was categorized as ≤9, 10–12, ≥13 years, or unknown. Similar to previous studies^43,44^, we used lifetime chronic obstructive pulmonary disease (COPD) as a proxy from smoking (ICD codes in Supplementary Table 2), but as 50% of life-long smokers may develop COPD^45^ and 25–45% of COPD patients have never smoked^46^, we also included a simulated smoking variable (see below). Comorbidities were ascertained from the Patient Register from 1964 up to the index date, and further weighted and categorized into three groups (0, 1–2, or ≥3 points) according to Deyo’s modification of the Charlson’s Comorbidity Index^47^ (ICD codes in Supplementary Table 2).

Cohort study in the Swedish Twin Registry: Age at the interview was categorized as ≤59, 60–69, 70–79, or ≥80 years. Information on educational attainment (≤9, 10–12, >12 years, or unknown), smoking status (ever or never), and alcohol consumption in the past month (yes or no) was retrieved from the telephone interviews. Via linkage to the Patient Register, we created the same variable as in the nested case-control study to quantify comorbidities before the end of follow-up.

### Statistical analysis

In the nested case-control study, the associations between IBS and PD risk were expressed as odds ratios (ORs) with 95% confidence intervals (CIs) estimated from conditional logistic regression. We performed analyses in two steps: first, conditional on sex and birth year matched sets, and second, additionally adjusted for country of birth, educational attainment, COPD, and comorbidity index. We analyzed the IBS in relation to PD risk during different time periods before the index date (<5, ≥5, <10, ≥10 years before). In addition, we conducted three sub-analyses, including interaction terms between IBS and sex, age at index date (≤69, 70–79, ≥80), and age at IBS diagnosis (<50, ≥50), respectively. Individuals with missing covariates were excluded from the analysis.

In the cohort study based on the Swedish Twin Registry, we estimated hazard ratios (HRs) with 95% CIs for PD from Cox proportional hazard models using attained age as underlying time scale. We used robust standard error to handle the relatedness within twin pairs. We examined the association first adjusted for attained age and sex, and secondly accounting for educational attainment, smoking, alcohol consumption, and comorbidity index. Individuals with missing covariates were excluded from the analysis. The comorbidity index was treated as a time-varying covariate. Comparisons within twin pairs and analyses of the temporal relationship were precluded due to small numbers. The proportional hazard assumption was examined using Schoenfeld residuals^48^ and tested using the Grambsch and Therneau’s method^48^. We did not note any violation of the proportional hazard assumption. Standardized survival functions of PD were estimated and plotted for IBS and non-IBS individuals using regression standardization over covariates’ distributions after fitting the multivariable adjusted Cox model^49^. In the absence of unmeasured confounding, these standardized survival curves have causal interpretations as representing the counterfactual scenarios where everybody versus nobody is exposed to IBS.

### Sensitivity analysis

To control for constipation and to better control for smoking, we included simulated variables of constipation (yes/no) and smoking (ever/never) in the above-mentioned multi-variable adjusted conditional logistic regression.

We simulated a binary constipation variable and adjusted for it in a two-step sensitivity analysis. First, we randomly assigned each individual with a binary constipation level from a Bernoulli distribution, with prevalence of 47% for IBS patients and 30% for non-IBS individuals. These prevalence rates were estimated from the SALT study in the Swedish twins (described in the main text) and used as proxies for constipation prevalence in the Swedish total population. Information about constipation in the twins were collected using the question “Have you ever had a recurrent problem of constipation (usually <3 bowel movements per week)?”. In the second step, we re-fitted a multivariable adjusted conditional logistic model including the same variables as in the main analysis but with additional adjustment for the simulated constipation variable. In this model, we fixed the log (OR) of constipation on PD risk to log (2.27) based on results from a meta-analysis^50^. Thus, the second step estimated the OR for IBS diagnosis on PD rate adjusted for constipation. To reduce the uncertainty of the estimate from the random generation of constipation level, we repeated the two steps 100 times and subsequently averaged the 100 estimates to produce a final estimate for IBS on the risk of PD. We adjusted for a simulated smoking variable in the same way. In the first step, we simulated a binary variable of ever or never smoking for each individual with prevalence of 63.1% in IBS patients and 58.3% in non-IBS participants estimated from the SALT study in the Swedish twins. In the second step, we fixed the log OR of smoking on PD risk to log (0.64) based on a meta-analysis^51^. CIs for the estimated ORs adjusted for constipation and smoking were obtained using 100 bootstrap replications of the entire procedure.

We calculated the *E*-value to estimate the minimum strength of association that an unmeasured confounder would need to have with both IBS and PD to justify that the observed association is entirely non-causal, with a large *E*-value indicating more robust results^52^. Second, to examine the influence of potential surveillance bias, i.e. that patients with IBS had more frequent hospital admissions than those without and therefore were more likely to receive PD diagnosis, we further adjusted for total number of hospital visits in quartiles (i.e. 0–1, 2–4, 5–9, and ≥10 visits before the index date). Third, given the higher PPV for PD primary inpatient diagnosis^34^, we also conducted additional analyses restricting the PD definition to primary diagnosis alone.

We used Stata 15 and SAS 9.4 for statistical analyses with two-sided alpha of 0.05. Standardized survival curves were estimated and plotted in R 3.4.1 using the stdReg package^49^.

### Standard protocol approvals, registrations, and participant consents

The study was approved by the Regional Ethics Review Board in Stockholm. The study based on registry-based health care data does not require informed consent from all study participants.

### Reporting summary

Further information on research design is available in the Nature Research Reporting Summary linked to this article.

## Supplementary information

Supplemental material

Reporting Summary Checklist

## Data Availability

The data analyzed in this study were obtained from the Swedish National Board of Health and Welfare and Statistics Sweden. We are not allowed to make the data publicly available according to the Swedish privacy laws. Request to access these data should be directed to the Swedish National Board of Health and Welfare and Statistics Sweden upon an ethical approval from a regional ethics review board.

## Code availability

Codes for data analysis in this study are available upon request.
