# Supplementary material for: Irritable bowel syndrome and Parkinson’s disease risk: register-based studies
Source: NPJ Parkinsons Dis. 2021 Jan 5;7:5. doi: 10.1038/s41531-020-00145-8 (PMC7785733; doi:10.1038/s41531-020-00145-8)
Supplement: Supplementary file 1 — Supplemental material [file 41531_2020_145_MOESM1_ESM.pdf]

Supplementary material

Supplementary Table 1 ICD codes for identification of inflammatory bowel disease, colorectal cancer and celiac disease.

Diagnosis	ICD-7<br>up to 1968	ICD-8<br>1969-1986	ICD-9<br>1987-1996	ICD-10<br>1997 onward
**Inflammatory bowel disease**				
Ulcerative colitis (UC)	572.20, 572.21	563.10, 563.99, 569.02	556	K51
Crohn's disease (CD)	572.00, 572.09	563.00	555	K50
Unclassified (IBD-U)	UC+CD	UC+CD	UC+CD	UC + CD, or K52.3
**Colorectal cancer**	153,154	153,154	153,154	C18,C19,C20,C21
**Celiac disease**	286.00	269.00	579A	K900

Supplementary Table 2 ICD codes for identifying comorbidity

	ICD-7<br>up to 1968	ICD-8<br>1969-1986	ICD-9<br>1987-1996	ICD-10<br>1997 onward
**- Myocardial infarction**	420.1	410, 411	410.x, 412.x	I21.x, I22.x, I25.2
**- Congestive heart failure**	434.1, 434.2	427.0, 427.1,428,782.4	428	I09.9, I11.0, I13.0, I13.2, I25.5, I42.0, I42.5-I42.9, I43.x, I50.x, P29.0
**- Peripheral vascular disease[a]**	450.x, 451, 453.3, 455	441, 443.9, 445	441,443.9,785.4, V43.4	I70.x, I71.x, I73.1, I73.8, I73.9, I77.1, I79.0, I79.2, K55.1, K55.8, K55.9, Z95.8, Z95.9
**- Cerebrovascular disease**	330-334	430-438	430.x-438.x	G45.x, G46.x, H34.0, I60.x-I69.x
**- Chronic obstructive pulmonary disease (COPD)**	502	491, 492	491,492,496	J41-J44
**- Other chronic pulmonary disease[b]**	523-524	515-517	500-505, 506.4	J60.x-J67.x, J68.4, J70.x, J70.3
**- Rheumatologic disease**	710.0, 726.3, 727<br>722.x, 525,	734.0, 734.1, 716.1, 712.1-712.5, 515.9, 717.9	710.0, 710.1, 710.4, 714.0-714.2, 714.81, 725	M05.x, M06.x, M31.5, M32.x-M34.x, M35.1, M35.3, M36.0
**- Osteoarthritis**	723	713	715	M15-M19
**- Dementia**	304, 305, 308	290, 309, 293.4	290	F00.x-F03.x, F05.1, G30.x, G31.1
**- Hemiplegia**	357, 351, 352	344.1. 344.2	344.1, 342	G04.1, G11.4, G80.1, G80.2, G81.x, G82.x, G83.0-G83.4, G83.9
**- Diabetes**	260.09 (only in Swedish ICD)	250	250.0, 250.1, 250.2, 250.3,250.7	E10.0, E10.1, E10.6, E10.8, E10.9, E11.0, E11.1, E11.6, E11.8, E11.9, E12.0, E12.1, E12.6, E12.8, E12.9,

				E13.0, E13.1, E13.6, E13.8, E13.9, E14.0, E14.1, E14.6, E14.8, E14.9
**- Diabetes with end organ damage**	260.20-260.99 (only in Swedish ICD)	250,01-250,05; 250,07-250, 09 (only in Swedish ICD)	250.4-250.6	E10.2-E10.5, E10.7, E11.2-E11.5, E11.7, E12.2-E12.5, E12.7, E13.2-E13.5, E13.7, E14.2-E14.5, E14.7
**- Moderate or severe renal disease**	592, 593, 603,792	582,583, 584, 593.2, 792, 593.0	582.x, 583-583.7, 585, 586, 588-588.9	I12.0, I13.1, N03.2-N03.7, N05.2-N05.7, N18.x, N19.x, N25.0, Z49.0-Z49.2, Z94.0, Z99.2
**- Mild liver disease**	581,583	571.x, 573	571.2, 571.4, 571.5, 571.6	B18.x, K70.0-K70.3, K70.9, K71.3-K71.5, K71.7, K73.x, K74.x, K76.0, K76.2-K76.4, K76.8, K76.9, Z94.4
**- Moderate or severe liver disease**	581,583,462,1	571.x, 573, 456.0	572.2, 572.3, 572.4, 572.8, 456.0-456.21	I85.0, I85.9, I86.4, I98.2, K70.4, K71.1, K72.1, K72.9, K76.5, K76.6, K76.7
**- Ulcer disease**	540, 541, 542	531-534	531-534	K25.x-K28.x
**- Any malignancy, including leukemia and lymphoma**	140-155, 157-164, 170-204	140-195, 200-209	140-172.9, 174-195.8, 200-208.9	C00.x-C26.x,C30.x-C34.x, C37.x-C41.x, C43.x, C45.x-C58.x, C60.x-C76.x, C81.x-C85.x, C88.x, C90.x-C97.x
**- Metastatic cancer**	156,163, 165	196-199	196,197,198,199.0, 199.1	C77.x- C80.x
**- HIV/AIDS**	not applicable	not applicable	042, 043, 044	B20.x-B22.x, B24.x

a. Also included surgery codes: 8884, 8885, 8815, 8816, 8817, 8818, 8886, 8887, 8825, 8826, 8827, 8828 from the Swedish Classification of Operations and Major Procedures. b. Other chronic pulmonary diseases including pneumoconiosis, and chronic respiratory conditions due fumes and vapors.

Supplementary Table 3 Irritable bowel syndrome diagnosis and risk of Parkinson's disease in nationwide nested case-control study: sensitivity analyses adjusting for simulated smoking status and constipation

	**OR (95% CI)[a]**	**OR (95% CI)[b]**	**Approximate RR / E-value[c] (95% CI lower band)[d]**
**Non IBS**	1	1	1
**IBS**	1.47 (1.30-1.66)	1.24 (1.10-1.40)	1.29 / 1.89 (1.64)

a: model conditional on sex and birth year matching pairs, country of birth, highest achieved education, COPD, comorbidity index, *simulated smoking status*; b. model conditional on sex and birth year matching pairs, country of birth, highest achieved education, COPD, comorbidity index, *simulated constipation status*, c. E-value gauges the robustness of the results to unmeasured confounding. A large E-value indicates more robust results. An E-value of 1.89 indicates that an unmeasured confounder associated with both IBS and PD by the relative risk of 1.89 each would explain away an approximate RR of 1.29 (equivalent to an OR of 1.44, CI: 1.27-1.63). d: The 95% CI lower band of the E-value (i.e. 1.64) was calculated for the 95% CI lower band of 1.27 for the IBS and PD association.

Supplementary Table 4 Irritable bowel syndrome diagnosis and risk of Parkinson's disease in nationwide nested case-control study: sensitivity analyses restricting to primary PD register diagnosis and adjusting for number of hospital visits

	Restricted to primary PD diagnosis		Adjusting for number of hospital visits	
	**PD/control [a]**	**OR (95% CI)[a]**	**PD/control[b]**	**OR (95% CI)[b]**
**Non IBS**	29,106/873,904	1	56311/1,691,716	1
**IBS**	128/3,116	1.27 (1.07-1.52)	253/5,204	1.23 (1.08-1.40)
Years before index date				
<5	48/1,339	1.09 (0.82-1.46)	93/2,105	1.12 (0.91-1.38)
≥5	80/1,777	1.41 (1.13-1.77)	160/3,099	1.31 (1.11-1.53)
<10	88/2,070	1.30 (1.05-1.61)	166/3,331	1.27 (1.08-1.48)
≥10	40/1,046	1.21 (0.88-1.66)	87/1,873	1.17 (0.94-1.45)
Age at IBS diagnosis, years				
<50	20/572	1.09 (0.70-1.71)	28/766	0.93 (0.63-1.35)
≥50	108/2,544	1.31 (1.08-1.59)	225/4,438	1.28 (1.12-1.47)
**Stratified by Sex**				
Male	44/1,102	1.23 (0.91-1.67)	89/1,789	1.27 (1.02-1.57)
Female	84/2,014	1.29 (1.04-1.61)	164/3,415	1.21 (1.04-1.42)
**Stratified by age on the index date, years**				
<70	58/1,242	1.45 (1.11-1.88)	80/1,538	1.29 (1.03-1.62)
70-79	51/1,321	1.19 (0.90-1.58)	101/2,184	1.17 (0.95-1.42)
≥80	19/553	1.08 (0.68-1.71)	72/1,482	1.27 (1.00-1.61)

a. restricted to *primary PD cases* and controls, n=906,254, conditional on sex and birth year matching pairs, country of birth, highest achieved education, chronic obstructive pulmonary disease (COPD), comorbidity index. No statistically significant interaction was found between IBS and age at IBS diagnosis (*p for interaction = 0.46)*. b. entire nested case-control study n=1,753,484, conditional on sex and birth year matching pairs, country of birth, highest achieved education, COPD, comorbidity index, *number of hospital visits*. No statistically significant interaction was found between IBS and age at IBS diagnosis (*p for interaction = 0.11)*.
