# Supplementary material for: Irritable bowel syndrome and Parkinson’s disease risk: register-based studies
Source: NPJ Parkinsons Dis. 2021 Jan 5;7:5. doi: 10.1038/s41531-020-00145-8 (PMC7785733; doi:10.1038/s41531-020-00145-8)
Supplement: Supplementary file 2 — Reporting Summary Checklist [file 41531_2020_145_MOESM2_ESM.pdf]

nature research

Corresponding author(s): NPJPARKD-00427R1

Last updated by author(s): Nov 1, 2020

Reporting Summary

Nature Research wishes to improve the reproducibility of the work that we publish. This form provides structure for consistency and transparency in reporting. For further information on Nature Research policies, see our Editorial Policies and the Editorial Policy Checklist.

Statistics

For all statistical analyses, confirm that the following items are present in the figure legend, table legend, main text, or Methods section.

n/a	Confirmed	
☐	☒	The exact sample size (*n*) for each experimental group/condition, given as a discrete number and unit of measurement
☒	☐	A statement on whether measurements were taken from distinct samples or whether the same sample was measured repeatedly
☐	☒	The statistical test(s) used AND whether they are one- or two-sided<br>*Only common tests should be described solely by name; describe more complex techniques in the Methods section.*
☐	☒	A description of all covariates tested
☒	☐	A description of any assumptions or corrections, such as tests of normality and adjustment for multiple comparisons
☐	☒	A full description of the statistical parameters including central tendency (e.g. means) or other basic estimates (e.g. regression coefficient) AND variation (e.g. standard deviation) or associated estimates of uncertainty (e.g. confidence intervals)
☐	☒	For null hypothesis testing, the test statistic (e.g. *F*, *t*, *r*) with confidence intervals, effect sizes, degrees of freedom and *P* value noted<br>*Give P values as exact values whenever suitable.*
☒	☐	For Bayesian analysis, information on the choice of priors and Markov chain Monte Carlo settings
☒	☐	For hierarchical and complex designs, identification of the appropriate level for tests and full reporting of outcomes
☒	☐	Estimates of effect sizes (e.g. Cohen's *d*, Pearson's *r*), indicating how they were calculated

Our web collection on statistics for biologists contains articles on many of the points above.

Software and code

Policy information about availability of computer code

Data collection	n/a. The data analyzed in this study were obtained from the Swedish National Board of Health and Welfare and Statistics Sweden. We are not allowed to make the data publicly available according to the Swedish privacy laws. Request to access these data should be directed to the Swedish National Board of Health and Welfare and Statistics Sweden upon an ethical approval from a regional ethics review board.
Data analysis	SAS and Stata codes for data analysis are available upon request.

For manuscripts utilizing custom algorithms or software that are central to the research but not yet described in published literature, software must be made available to editors and reviewers. We strongly encourage code deposition in a community repository (e.g. GitHub). See the Nature Research guidelines for submitting code & software for further information.

Data

Policy information about availability of data

All manuscripts must include a data availability statement. This statement should provide the following information, where applicable:

- Accession codes, unique identifiers, or web links for publicly available datasets
- A list of figures that have associated raw data
- A description of any restrictions on data availability

The data analyzed in this study were obtained from the Swedish National Board of Health and Welfare and Statistics Sweden. We are not allowed to make the data publicly available according to the Swedish privacy laws. Request to access these data should be directed to the Swedish National Board of Health and Welfare and Statistics Sweden upon an ethical approval from a regional ethics review board.

Field-specific reporting

Please select the one below that is the best fit for your research. If you are not sure, read the appropriate sections before making your selection.

☒ Life sciences ☐ Behavioural & social sciences ☐ Ecological, evolutionary & environmental sciences

For a reference copy of the document with all sections, see nature.com/documents/nr-reporting-summary-flat.pdf

Life sciences study design

All studies must disclose on these points even when the disclosure is negative.

Sample size	Nationwide nested case-control study. Based on the Population and Housing Census in 1980, we conducted a nested case-control study in the total population of Sweden, who were born between 1889 and 1980 without previous PD diagnosis, and lived in Sweden on January 1, 1987 (N = 7,557,897). PD patients were identified between January 1, 1987 and December 31, 2010 by linkage to the Patient Register. For each PD patient, we randomly selected 30 controls, who were alive and living in Sweden, currently without PD diagnosis, individually matched to the case by sex and year of birth, on the date of PD diagnosis. The date of PD diagnosis was hereafter referred to as the index date for both PD cases and controls. In total, 56,564 PD cases and 1,696,920 controls were included in the nested case-control study.<br><br>Cohort study in the Swedish Twin Registry. The cohort study was based on all twins who responded to the SALT interview carried out in 1998-2002 (N= 44,919). Twins who discontinued participation with missing information on linkage or the date of emigration, or errors on date of death (N=69), or had a PD diagnosis identified from the Patient Register or by self-report before or at the interview (N=96), or had missing information on IBS at the interview (N=529) were excluded. In total, the study population included 44,225 twins, who were followed from the date of interview until date of PD diagnosis, death, emigration out of Sweden, or December 31, 2016, whichever occurred first.
Data exclusions	Nationwide nested case-control study. We included who were born between 1889 and 1980 without previous PD diagnosis, and lived in Sweden on January 1, 1987.<br>Cohort study in the Swedish Twin Registry. Twins who discontinued participation with missing information on linkage or the date of emigration, or errors on date of death (N=69), or had a PD diagnosis identified from the Patient Register or by self-report before or at the interview (N=96), or had missing information on IBS at the interview (N=529) were excluded.
Replication	n/a
Randomization	n7a
Blinding	n/a

Reporting for specific materials, systems and methods

We require information from authors about some types of materials, experimental systems and methods used in many studies. Here, indicate whether each material, system or method listed is relevant to your study. If you are not sure if a list item applies to your research, read the appropriate section before selecting a response.

Materials & experimental systems

n/a	Involved in the study	
☒	☐	Antibodies
☒	☐	Eukaryotic cell lines
☒	☐	Palaeontology and archaeology
☒	☐	Animals and other organisms
☐	☒	Human research participants
☒	☐	Clinical data
☒	☐	Dual use research of concern

Methods

n/a	Involved in the study	
☒	☐	ChIP-seq
☒	☐	Flow cytometry
☒	☐	MRI-based neuroimaging

Human research participants

Policy information about studies involving human research participants

Population characteristics	The study used the data through linkage of several Swedish National Registries.
Recruitment	The study based on registry-based health care data does not require informed consent from all study participants.
Ethics oversight	The study was approved by the Regional Ethics Review Board in Stockholm. The study based on registry-based health care data does not require informed consent from all study participants.

Note that full information on the approval of the study protocol must also be provided in the manuscript.
